# Revealing the Influence of Material Properties of Shaped Charge Liner on Penetration Performance via Numerical Simulation and Machine Learning

**DOI:** 10.3390/ma18122742

**Published:** 2025-06-11

**Authors:** Yan Wang, Jinxu Liu, Xingwei Liu, Xinya Feng, Yifan Du, Jie Cao

**Affiliations:** 1 School of Materials Science and Engineering, Beijing Institute of Technology, Beijing 100081, China; wangyanbit@126.com (Y.W.); xinya_feng@126.com (X.F.); duyifan2025@126.com (Y.D.); wy2055917886@163.com (J.C.); 2 China National Key Laboratory of Science and Technology on Materials Under Shock and Impact, Beijing Institute of Technology, Beijing 100081, China

**Keywords:** shaped charge, metal jet, penetration mechanism, numerical simulation, machine learning

## Abstract

The metallic shaped charge liner (SCL) is widely utilized in the defense industry, oil perforation, cutting, and other industrial fields due to the powerful penetration performance. However, quantitative law and underlying mechanisms of material properties affecting SCL penetration performance are unclear. Based on the real and virtual material properties, by combining numerical simulation with machine learning, the influence of material properties on SCL penetration performance was systematically studied. The findings in the present work provided new insights into the penetration mechanism and corresponding influencing factors of the metal jet. It indicated that penetration depth was dominated by the melting point, specific heat, and density of the SCL materials rather than the conventionally perceived plasticity and sound velocity. Average perforation diameter was dominated by the density and plasticity of the SCL materials. Particularly, the temperature rise and thermal softening effect of the SCL controlled by the melting point and specific heat have a significant effect on the “self-consumption” of the metal jet and further on the penetration ability. Additionally, the density of the SCL influences the penetration depth deeply via dynamic pressure of the jet, but the influence of density on penetration depth decreases with the increase in density. The correlation between the key properties and penetration performance was obtained according to a quadratic polynomial regression algorithm, by which the penetration potential of SCL materials can be quantitatively evaluated. Overall, the present study provides a new SCL material evaluation and design method, which can help to expand the traditional penetration regime of the SCL in terms of the penetration depth and perforation and is expected to be used for overcoming the pierced and lateral enhancement trade-off.

## 1. Introduction

Relying on a formed high-speed metal jet, the metallic SCL can readily penetrate hard targets such as armor, rock, and concrete at extremely high front-end speeds (about 6–10 km s^−1^) driven by explosives [1,2,3,4,5], which are widely used in the defense industry, oil perforation, cutting, and other industrial fields due to its powerful penetration capabilities [6,7,8,9,10]. Many studies have evaluated the penetration capacity of SCL structures (such as hypervelocity shaped charge [11], double-layer [12,13], spin-compensation structure [14], bell and bi-conical shape liner [15,16,17,18], W type [19], and so on) or materials (such as titanium alloy [2,20], sintered copper [1], Al/PTFE [7,21,22,23], copper–tungsten alloy [24,25,26], high-entropy alloy [27], reactive metal [28,29], and so on). Traditional penetration mechanics theory holds that the penetration depth of the SCL is proportional to the square root of density, and the plasticity of SCL material affects jet formation [11,15,30,31,32]. However, the effects of basic physical properties (such as yield strength, strain rate hardening ability, thermal softening ability, sound velocity, specific heat, melting point, etc.) on the penetration performance of SCL materials are lacking in quantitative studies. In fact, based on the regulation of SCL materials, the penetration performance of the shaped charge has stagnated over the past decade. The possibility of other key properties of SCL material affecting the penetration performance, as well as their influence mechanisms on the jet formation and interaction with the target, has remained unknown.

By changing a specific physical property, the relationship between the penetration performance and the physical properties of the SCL can be studied via numerical simulation. The jet forming driven by explosives is a rapid and complex process, accompanied by the deformation and temperature rise of the SCL [33]. The formation and penetration process of the metal jet can be clearly observed by numerical simulation, which is helpful to analyze the influence of material properties on jet penetration behavior. Numerous studies have investigated the jet formation and penetration behaviors of various SCLs using numerical simulation methods. The primary algorithms employed in these simulations include the Euler algorithm [15,21], the arbitrary Lagrange–Euler (ALE) algorithm [34,35], and the smoothed particle hydrodynamics (SPH) algorithm [7,36]. For instance, Elshenawy et al. [15] developed an Euler-based model for shaped charge jet formation using AUTODYN software. Their model provided clear jet morphology data, and the simulation results exhibited strong agreement with experimental findings. Similarly, Hu et al. [35] utilized the ALE algorithm to simulate the formation of explosively formed projectiles (EFPs). Notably, their study employed the Lagrange algorithm to model concrete targets and established fluid structure interaction to analyze the interaction between ALE-modeled EFPs and the targets. The simulations demonstrated that this approach accurately captures both EFP formation and concrete target damage. Furthermore, Chen et al. [36] applied the SPH algorithm to model jet formation and penetration. Their results confirmed that SPH-based simulations effectively reproduce the process of jet penetration into ceramic targets, with close alignment between numerical predictions and experimental data. Therefore, numerical simulation is an efficient and economical method to study the penetration behavior of a large number of different SCLs with different properties.

The formation and penetration process of a metal jet, as a high-strain-rate state, cannot be effectively described by the Lagrange algorithm due to the numerical instability of this algorithm under high-strain conditions [3,12]. The traditional Euler method relies on high-precision structured grids to calculate the movement of the boundary, necessitating an extremely high grid density in the models of jet formation and penetration process [37]. The arbitrary Lagrange–Euler (ALE) method overcomes the above drawbacks by introducing reference grids, making it a robust approach to analyze the dynamic response of structures subjected to explosive loading [38]. The penetration process is a typically fluid structure interaction (FSI) condition. The penalty function is employed to define the contact between ALE material and Lagrange algorithm elements [38,39]. In the complex scenario where an explosion impact causes structural deformation while fluids are involved, the ALE part is used to handle the calculations related to fluids, and the Lagrange part is used to deal with the calculations related to structures. And the penalty function can define the contact and interaction between them, making the whole simulation more in line with the actual physical laws. In addition, the process of jet formation and penetration can be regarded as an ideal process of symmetrically rotating around the penetration direction. Therefore, the ALE-FSI model can be used in analyzing the jet formation and penetration process of SCL effectively [40].

Machine learning (ML) methods can leverage large-scale experimental or simulation databases to analyze multi-dimensional parameter relationships, enabling the identification of key factors influencing shaped charge performance or providing iteratively optimized design solutions. Consequently, ML-driven parameter optimization has emerged as a promising approach for advancing SC structural and material design in recent years. For instance, Sterbentz et al. [41] employed artificial neural networks (ANNs) to optimize the initiation position and delay time for a specific SC configuration, resulting in increased jet head velocity and enhanced mass concentration at the jet’s front end. Similarly, Zhao et al. [42] developed a hybrid optimizer by integrating the XGBoost algorithm with multi-model fusion optimizer (MFO) and applied it to a shaped charge liner (SCL) design, achieving a remarkable 269% improvement in penetration depth. Various physical properties of SCL materials are likely coupled in influencing jet formation and penetration performance. Machine learning algorithms offer an efficient approach to identify the key governing parameters from these complex interdependencies [41,42]. In order to reduce redundant calculation and multi-variable interference, ensuring the prediction precision and efficiency of machine learning, a virtual material method, adaptive boosting (Adaboost) regression algorithm, and regression equation were performed in the present work. Based on the regression equation of the key properties and penetration performance obtained from the regression algorithm, the penetration potential of SCL materials can be evaluated quantitatively, and modification targets of SCL materials based on application scenarios can be clarified [43,44,45]. Compared with previous SCL material design research, by using the virtual material method and combining numerical simulation with machine learning, the wide-area performance range of common metal materials can be examined more extensively, thereby providing representative and widely applicable SCL material design criteria.

In this study, common metal materials with very different properties were used as reference materials to evaluate the penetration performance of the SCL using the ALE-FSI model. To expand the material library, the virtual material method is used in this work based on changing a single physical parameter of the reference material. A material library composed of the extension and reference materials was then used for numerical simulation to obtain a database of the relationships between material properties and penetration performance. The effects of various physical properties on penetration and perforation were decoupled and analyzed by the Adaboost algorithm based on a regression tree. The relative importance ranking indicating the key properties of material physical properties that affected the penetration ability of the SCL was given. Based on the key properties given by the Adaboost algorithm, a set of formulas for quantitative evaluation of penetration depth and perforation diameter of SCL materials were given by a quadratic polynomial regression algorithm. The properties of a kind of zirconium alloy and a kind of titanium alloy were introduced for the evaluation formula, and related experiments were performed to verify the effectiveness of the formula. Through the post-processing for numerical simulation results, the influence mechanism of the key properties on jet penetration behavior was analyzed in detail. A new dynamic and thermodynamic theories of jet behavior based on material properties were proposed.

## 2. Research Methodology

### 2.1. Numerical Simulation Method

A two-dimensional symmetric ALE-FSI model whose average element size is 0.5 mm was established to analyze the jet formation and penetration process of the SCL using LS-DYNA (v2021 R1). This model was not only used to obtain quantitative relationships between material properties and penetration performance but also to analyze the influence mechanism of SCL density on penetration and perforation [46].

The computational mesh of the ALE algorithm is not attached to fluid particles. In order to observe and analyze the local material movement and temperature change of the SCL in the process of jet formation and penetration, a model based on the smoothed particle hydrodynamics (SPH) algorithm with the same charge structure of the ALE-FSI model is established [47,48,49]. The SPH model is convenient for post-processing and secondary development based on Python (v3.8) and LS-Reader (v0.1.47), which is conducive to the statistics and analysis of the influence of thermodynamic properties of material on the jet formation and penetration process [36,50,51].

The Johnson–Cook (J-C) model is considered suitable for describing extreme physical states of the metal jet involved in large-deformation, high-strain-rate, and high-temperature processes [52]. The model also contained the influence of strain rate and temperature on stress–strain relationships. The stress defined by the J-C model is represented as

(1) $$\sigma =\left(A+B{\varepsilon_{p}}^{n}\right)\left(1+C\ln{\dot{\varepsilon }}^{*}\right)\left(1-{T^{*}}^{m}\right),$$

where $\varepsilon_{p}$, ${\dot{\varepsilon }}^{*}$, and $T^{*}$ are equivalent plastic strain, dimensionless equivalent strain rate, and dimensionless temperature, respectively. A (initial yield strength), B (strain hardening coefficient), n (strain hardening index), C (strain rate hardening coefficient), and m (thermal softening index) are constitutive parameters of the J-C model [53,54].

The Mie–Grüneisen equation of state (EOS) is considered to be able to describe the physical state of metal materials well in high-pressure environments [55]. The pressure defined by the Mie–Grüneisen EOS is represented as

(2) $$P=\frac{\rho_{0}{C_{0}}^{2}\varepsilon \left(1-\frac{\gamma_{0}}{2}\right)}{{\left(1-S_{1}\varepsilon \right)}^{2}}+\gamma_{0}U,\;$$

where $\rho_{0}$ and $C_{0}$ mean the density and bulk sound speed of the material at ε = 0. $S_{1}$ is the slope of the shock Hugoniot in the sound speed (*vs*)–particle speed (*vp*) plane. $\gamma_{0}$ and U are the Grüneisen constant and the internal energy within the material, respectively [56,57].

The Jones–Wilkins–Lee (JWL) EOS can accurately depict the pressure, volume, and energy characteristics of the detonation products during the explosion pressure process [15,58,59]. The explosives used in both experiments and numerical simulations are Hexogen (RDX). The properties of RDX in simulation models described by the JWL EOS are presented in Table 1 [60,61].

**Figure 1:**
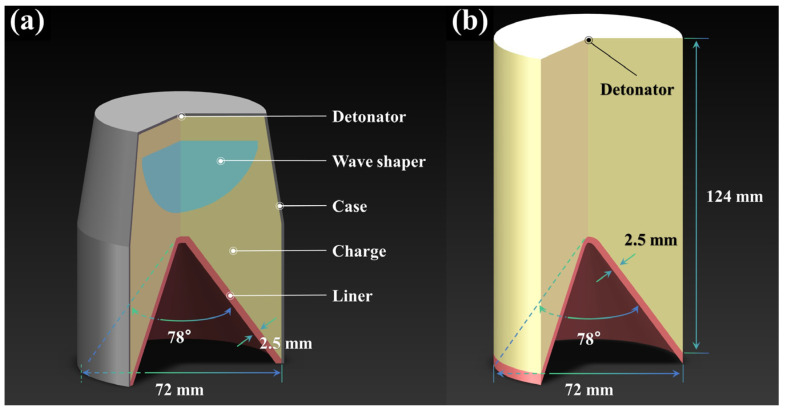
Structure of SC used for model validation and experiment verification (**a**), along with the Structure for numerical simulation of the materials library (**b**).

Existing research [2,20] utilized X-ray images to capture the instant of the jet formation of the Ti-17Al-29Nb alloy (named Ti Alloy) SCL and assessed the penetration capacity of this type of SCL. The experimental outcomes of Ti Alloy were employed for calibration and validation of the aforementioned established two-dimensional symmetric ALE-FSI model. The structure of the shaped charge is depicted in Figure 1a. The Ti Alloy was re-prepared to determine its J-C model and Mie–Grüneisen EOS, with the results presented in Table 2 and Table 3, respectively [2,62,63]. In a penetration capacity test, AISI 1045 steel (AISI 1045) was characterized using the J-C model in recent research [6,7,64]. A representative measurement result, as shown in Table 4 and Table 5, was employed to portray the target of the Lagrange algorithm in the ALE-FSI model [65,66].

$\rho_{0}$, $C_{0}$, $S_{1}$, and $\gamma_{0}$ from the Mie–Grüneisen EOS and A, B, n, C, and m from the J-C model as well as specific heat ($C_{V}$) and melting point ($T_{m}$) were selected to define SCL materials.

**Figure 2:**
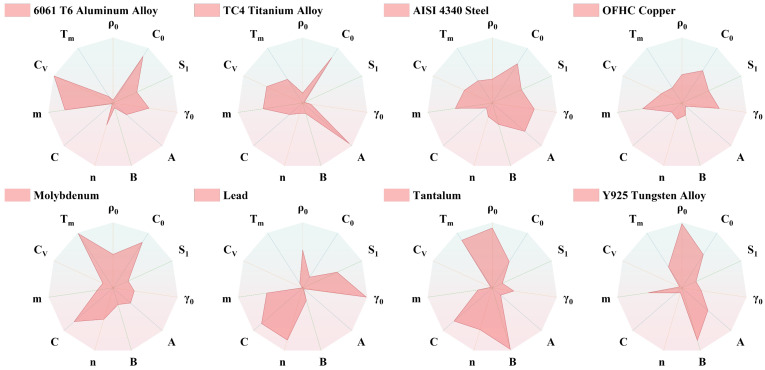
Radar chart of reference materials properties. There are significant differences in the properties of the eight reference materials, and there is no significant correlation between any two properties. These eight representative materials cover nearly the full range of properties found in common metal materials.

In order to comprehensively evaluate the influence of metallic materials’ properties on the penetration performance of the SCL, eight materials with diverse properties were selected as reference materials based on previous studies [67,68,69,70,71,72,73,74]. These materials include 6061-T6 aluminum alloy (6061-T6) [68,75], TC4 titanium alloy (TC4) [67,76,77], AISI 4340 steel (4340) [69,78], oxygen-free high-conductivity copper (OFHC Copper, Cu) [69,70], molybdenum (Mo) [71], lead (Pb) [72,79], tantalum (Ta) [73,80,81], and Y925 tungsten alloy (Y925) [74,82] (ordered by $\rho_{0}$ from smallest to largest). It should be noted that the materials involved in numerical simulations are abstracted models intended to consider the physical and mechanical properties of common metallic materials with significant property variations, without considering chemical reactions, atomic diffusion, or phase changes. For example, the boiling point of Pb is extremely low (about 2013 K), which may lead to the gasification of the jet, and the chemical reaction between tungsten and iron can lead to a severe reduction in penetration depth [83]. The properties of the eight reference materials are listed in Table 6. Radar charts (Figure 2) depict the properties of these eight materials, and each radar chart has the same range of axes. The comparison between radar charts demonstrates significant differences in properties with no significant correlations among the material properties.

The reference materials were expanded to provide a more comprehensive and detailed analysis how material properties influence the performance of the SCL. Because the eight reference materials almost cover the density range of metal materials, the density will not be extended in the extension material library. A total of 160 kinds of virtual materials were obtained by changing a single parameter of the reference material. The properties of the extension materials are described in Table S1 in Supporting Information Section S1. These 160 kinds of extension materials and eight kinds of reference materials together formed a material library for numerical simulation. Since the explosive mass in the shaped charge structure shown in Figure 1a is not sufficient to drive the SCLs of high-density metals of the material library, the shaped charge structure depicted in Figure 1b was employed to simulate the jet formation and penetration process of various SCL materials of the library. The explosive mass in this structure is sufficient to fully drive all kinds of simulated SCL materials. And center initiation was employed, too.

### 2.2. Machine Learning Method

The regression model was trained via machine learning techniques to explore the relationship between physical properties and simulation results within the material library. The simulation results generated by the ALE-FSI model are highly repeatable, but the database size is limited. With its present database characteristics, it is conducive for machine learning algorithms by employing weak supervision and robust regressors, thereby enabling the regression model to strike a balance between prediction accuracy and robustness. The database comprising the materials library and numerical simulation results was utilized in the adaptive boosting (Adaboost) algorithm based on the decision tree regressor. It facilitated the determination of the relative importance ranking of each material property concerning the penetration depth (Dep.) and the average perforation diameter (Dia.) of the SCL. The hyperparameters of the regression algorithm, such as max depth of the decision tree, number of estimators, and learning rate, have an impact on the accuracy and robustness of the regression model [84]. Through a random search of the hyperparameter grid, the hyperparameter combination that is most suitable for the current dataset can be found, and an Adaboost regression model that takes into account both accuracy and robustness can be obtained. Subsequently, the key properties, identified through the relative importance ranking, were subjected to a quadratic polynomial regression algorithm. This process yielded a comprehensive scoring formula for assessing the penetration and perforation capabilities of different metallic materials.

### 2.3. Experimental Verification Method

An SCL with medicated metal Zr is expected to enhance penetration ability and achieve energy release features [28]. The properties of the Zr, such as density, melting point, specific heat, mechanical properties, etc., can be adjusted by alloying. Based on the conclusions derived from numerical simulation and machine learning, an active metal material named Zr Alloy was fabricated using powder metallurgy. Employing the same structure and conditions of the shaped charge with Ti Alloy [9], the penetration and perforation capability of Zr Alloy were evaluated and compared with Ti Alloy by experiments.

**Figure 3:**
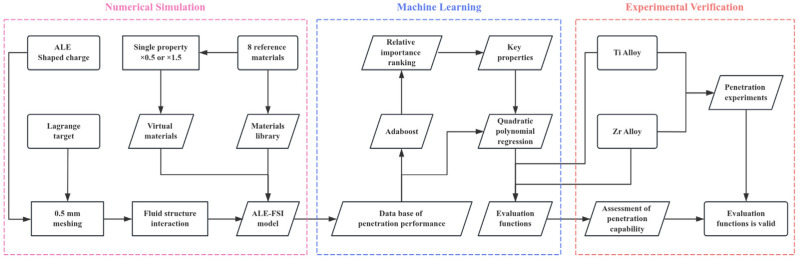
Flow chart of the research methodology.

The flow chart of the research methodology (numerical simulation, machine learning, and experimental verification) is shown in Figure 3.

## 3. Results of Simulation and Experiment

### 3.1. Simulation Model Verification

**Figure 4:**
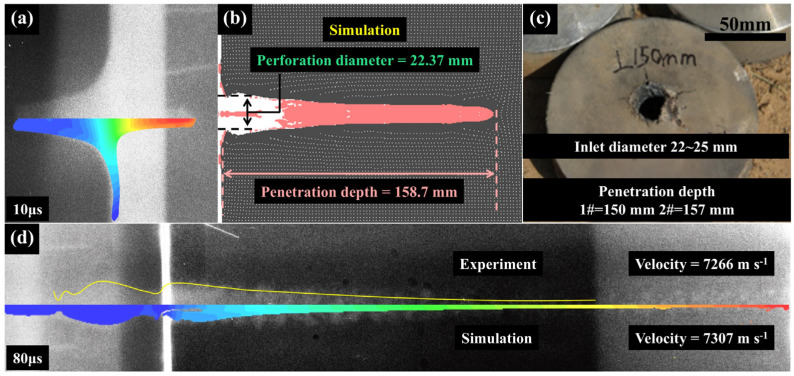
Comparison of Ti Alloy jet morphology between X-ray images and numerical simulation at 10 μs (**a**) and 80 μs (**d**), along with the penetration simulation performance (**b**) against experimental penetration results (**c**) of Ti alloy SCL.

The jet morphologies of the Ti Alloy SCL at 10 μs and 80 μs after detonation are shown in Figure 4a and Figure 4d, respectively. At 80 μs after detonation, the velocity of the jet head shown by X-ray was 7266 m s^−1^, while that of the jet head in simulation results was 7307 m s^−1^, with a difference of 0.5%. The jet morphologies, as described by the 2D symmetric ALE-FSI model, closely resemble those observed in the X-ray images at 10 μs and 80 μs after detonation. The penetration depth of Ti Alloy obtained by numerical simulation was 158.7 mm and the inlet diameter was 22.37 mm, while the penetration depth of the two repeated penetration trials was 150 mm and 157 mm, respectively, and the inlet diameter is between 22 and 25 mm. This correspondence is evident in Figure 4b,c, where the disparities in penetration depth and inlet diameter on the AISI 1045 target between the simulation and the experiment are within 5%. This suggests that the simulation model with a high convergence can accurately depict both the jet formation and the penetration process of the SCL on the AISI 1045 target.

### 3.2. Simulation Results and Discussion

**Figure 5:**
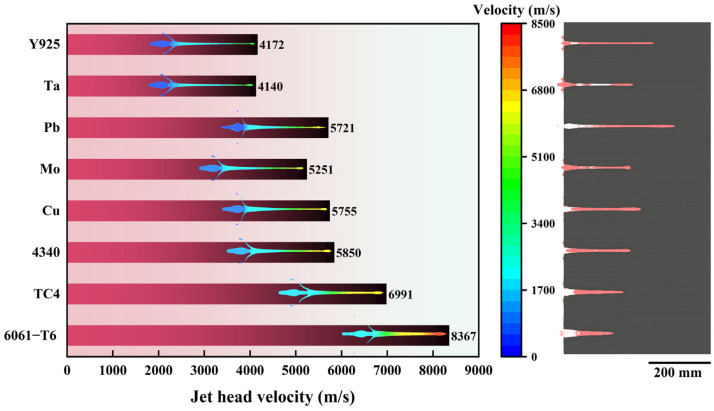
Jet head velocity and jet velocity distribution of reference materials at the moment of impact, along with the state of the AISI 1045 target after penetration.

**Figure 6:**
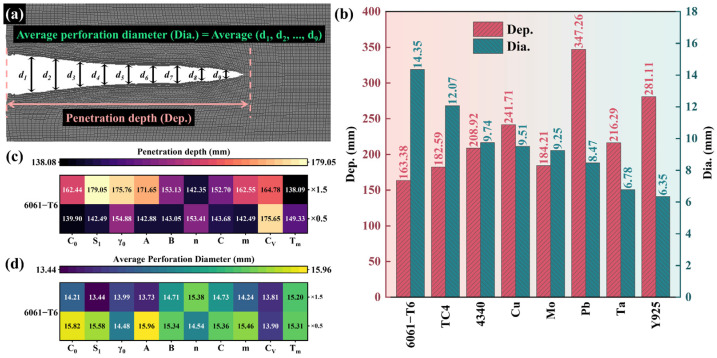
The measurement methods for the penetration depth (Dep.) and the average perforation diameter (Dia.) (**a**); The simulation results of reference materials (**b**); The Dep. of 6061-T6 Aluminum Alloy and its extension materials (**c**); The Dia. of 6061-T6 Aluminum Alloy and its extension materials (**d**).

Figure 5 shows the simulation results which depict the head velocity of the reference material at the moment of the jet impacting on the target and the subsequent target plate profile after penetration. The 6061-T6 jet, characterized by the lowest density among the listed materials, exhibits the highest velocity (8367 m s^−1^) but the lowest penetration depth (163.38 mm). The Pb jet demonstrates the deepest penetration (347.26 mm) among the reference materials. It is commonly believed that a jet head with a high velocity and density generally has a strong penetration depth. However, in the selected eight reference materials, there is no significant monotone correlation among jet head velocity, penetration depth, and material density. Among the four reference materials 6061-T6, TC4, 4340, and Cu a lower velocity of the metal jet and a higher penetration depth can be achieved in materials with a higher density. But, in the other four materials with higher density, Mo, Pb, Ta, and Y925, the monotonic relationship between density and jet velocity is broken. To quantify these observations, the measurement method depicted in Figure 6a was employed to determine the penetration depth and the average perforation diameter. The Dia. is the arithmetic average of the diameter of the entire perforation interior in the depth direction of ten equal points. The penetration depths and the average perforation diameters of the liners formed by the eight reference materials are illustrated in Figure 6b. The 6061-T6 jet with the lowest density in the reference materials formed the largest average perforation diameter (14.35 mm). In the eight reference materials, the average perforation diameters were negatively correlated with material density. Among the four metals with lower density, 6061-T6, TC4, 4340, and Cu, the Dep. increased with density, but in the four metals with higher density, Mo, Pb, Ta, and Y925, no such rule existed. This suggests that the influencing factors of the Dep. may be complex. Based on the material library expansion method given above, the simulation results of the penetration depths and the average perforation diameters of the reference materials and their extension materials are shown by heatmaps. As shown in Figure 6c,d, 6061-T6 aluminum alloy is taken as an example, and the values displayed within each cell represent the simulation results obtained by expanding the material. These were achieved via multiplying the single property of the column by the coefficients (×0.5 or ×1.5), respectively, based on the reference material. For example, based on the performance properties of 6061-T6, the extension material obtained via multiplying $C_{V}$ by 0.5 formed a penetration depth of 175.65 mm and an average perforation diameter of 13.90 mm. Here, the density was not involved in the expansion because there were large variations in the densities among the selected reference materials.

**Figure 7:**
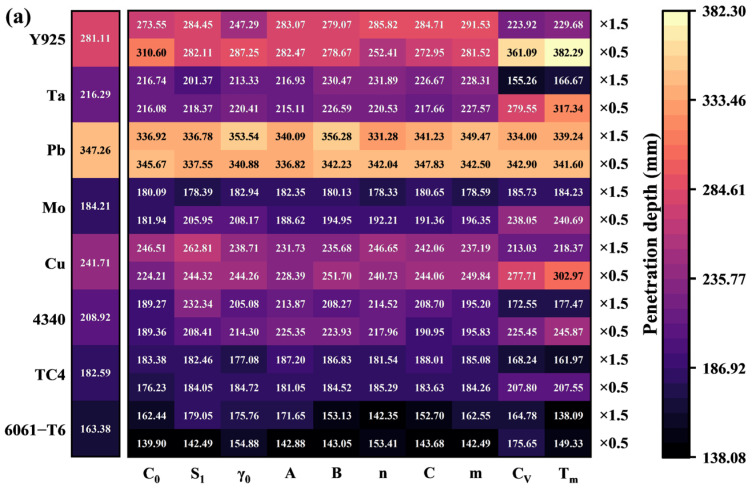

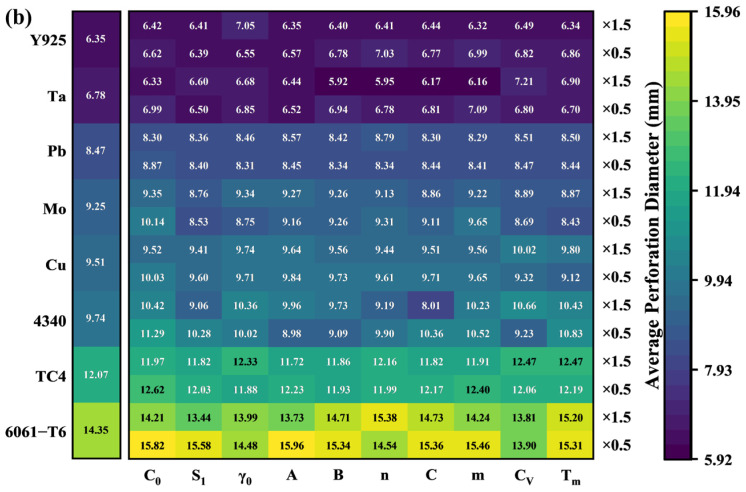
Heatmaps of the Dep. (**a**) and Dia. (**b**) simulation results for both reference and extension materials.

All simulation results of penetration depth and diameter from the material library are presented in the form of heatmaps, as shown in Figure 7. It can be found that the material extended by $T_{m}$ of Y925 multiplied by 0.5 achieves the largest Dep. of all simulation results, measuring 382.29 mm. The material extended by A of 6061-T6 multiplied by 0.5 achieves the largest Dia. of all simulation results, measuring 15.96 mm. It is noted that Pb and its extension materials formed a penetration depth of more than 300 mm. For other reference materials, the penetration depth increased with the decrease in $C_{V}$ and $T_{m}$.

Following this, to further investigate the relationship between material properties and penetration performance, the database formed by the numerical simulation results shown in Figure 7 and the corresponding material library was used for the Adaboost algorithm. This is helpful to decouple the mutually coupled material properties and find out the key material properties that significantly affect SCL penetration performance.

### 3.3. Results of Machine Learning Analysis

**Figure 8:**
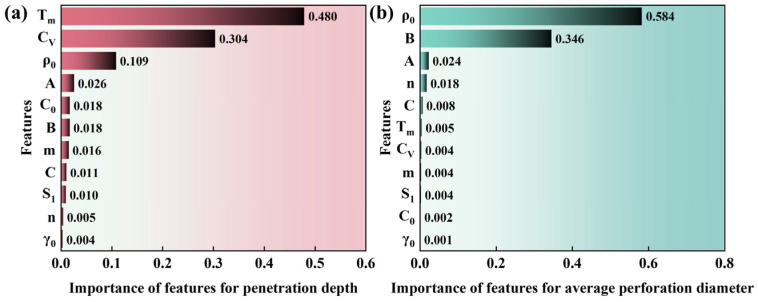
The relative importance ranking of material properties for Dep. (**a**) and Dia. (**b**).

The Adaboost regression model was trained on the database formed by the material library and simulation results for obtaining the relative importance ranking of all material properties. The Python code for the Adaboost algorithm to perform machine learning on these datasets is attached to Figure S1 in the Supporting Information. All the data is divided into a training set and test set, in which the test data was 30%. The hyperparameter grid is embedded in the algorithm to search for the optimal combination of hyperparameters. The parameter ranges corresponding to number of estimators, learning rate, maximum tree depth, minimum sample number of split nodes, and minimum sample number of leaf nodes are [50, 300], [0.01, 1.0], [3, 10], [2, 20], and [1, 10], respectively. The results show that when the number of estimators is 147, the learning rate is 0.36, the maximum tree depth is 9, the minimum sample number of split nodes is 3, and the minimum sample number of leaf nodes is 6, the regression effect of this algorithm on the Dep. database is the best. The R-square score of the training set is 0.97, and that of the test set is 0.90. The R-square score in k-fold cross-validation is 0.9000 (±0.0700), which means that there is no overfitting in the Dep. model. When the number of estimators is 51, the learning rate is 0.03, the maximum tree depth is 6, the minimum sample number of split nodes is 4, and the minimum sample number of leaf nodes is 8, the regression effect of this algorithm on the Dia. database is the best. The R-square score of the training set is 0.98 and that of the test set is 0.93. The R-square score in k-fold cross-validation is 0.9644 (±0.0105), which means that there is no overfitting in the Dia. model [85]. Based on the model determined by the above hyperparameters, the relative importance rankings of all material properties for the Dep. and the Dia. are shown in Figure 8a and Figure 8b, respectively. The influence of $T_{m}$ and $C_{V}$ on the penetration depths of liner materials is significantly stronger than that of other material properties, and their importance is 0.480 and 0.304, respectively. In combination with Figure 7, it can be found that $T_{m}$ and $C_{V}$ show a strong monotone negative correlation to the Dep. of each reference material except Pb, which means that reducing the $T_{m}$ and $C_{V}$ of the material can effectively improve the penetration depth of the SCL.

The impact of each physical property on the Dep. is non-monotonic and exhibits varying influence on different reference materials, possibly due to the coupling effects of diverse physical properties. For example, with the increase in the initial yield strength (A), the penetration depth of 6061-T6 was increased from 142.88 mm to 171.65 mm, but that of Mo was decreased from 188.62 mm to 182.35. For each reference material, the influence of $C_{V}$ and $T_{m}$ on the average perforation diameters is not as pronounced as that on the penetration depths. But $\rho_{0}$ and B have a significant advantage in their ability to influence the Dia. of the SCL.

Material properties with relative importance greater than 0.1 were selected as the key properties of the Dep. and the Dia., indicating that $T_{m}$, $C_{V}$, and $\rho_{0}$ were identified as the main influencing factors of the penetration depth, whereas $\rho_{0}$ and B are identified as the main influencing factors of the average perforation diameter. By quadratic polynomial regression of the key properties, the Dep. can be expressed as

(3) $$Dep.=16.20+\left\{\left[-4.65\times 10^{-3},-2.08\times 10^{5},3.44\right]+\left[T_{m},\;C_{V},\;\rho_{0}\right]\left[\begin{array}{ccc} {3.95\times 10}^{-7} & 444 & {-1.82\times 10}^{-4}\\ 0 & -3.83 & -2.60\times 10^{5}\\ 0 & 0 & -9.58\times 10^{-2} \end{array}\right]\right\}\times \left[\begin{array}{c} T_{m}\\ C_{V}\\ \rho \end{array}\right],\;$$

whose Pearson correlation coefficient is 0.9733, the *p*-value of Pearson’s correlation coefficient is 4.544 × 10^−108^, mean absolute error (MAE) is 1.040, and mean square error (MSE) is 1.900. The Dia. can be expressed as

(4) $$Dia.=1.72+\left\{\left[-9.87\times 10^{-2},-34.3\right]+\left[\rho_{0},B\right]\left[\begin{array}{cc} 2.12\times 10^{-3} & 2.30\\ 0 & -314 \end{array}\right]\right\}\times \left[\begin{array}{c} \rho_{0}\\ B \end{array}\right],$$

whose Pearson correlation coefficient is 0.9805, the *p*-value of Pearson’s correlation coefficient is 2.108 × 10^−119^, MAE is 0.038, and MSE is 0.003. This indicates that the predicted values given by the quadratic polynomial regression equations of the Dep. and the Dia. have a highly positive linear correlation with the true values, and the correlation is highly significant, which can be seen as a evaluation function to compare the penetration and perforation capabilities of materials (the g-cm-μs-K units were adopted, respectively).

The influence of shaped charge structure and explosive performance on penetration capability has been reported in previous studies [15,86]. It is important to acknowledge that factors such as shaped charge structure and explosive performance also influence penetration capability, which may affect the weighting assigned by machine learning algorithms or specific coefficients in regression equations.

The SCL geometry employed in this study falls within conventional design parameters, ensuring broad applicability of our findings. The RDX adopted is one of the most widely used SC explosives, and the charge mass is sufficient for the SCL of all materials. This standardized experimental configuration yields representative results that are both reliable and reproducible.

Importantly, while these specific parameters were chosen to isolate material effects, our integrated numerical simulation and machine learning framework maintains full adaptability for analyzing diverse SC configurations. The methodology’s flexibility allows for systematic investigation of alternative geometries, explosive types, and scaling effects while preserving the fundamental insights gained from this study.

**Figure 9:**
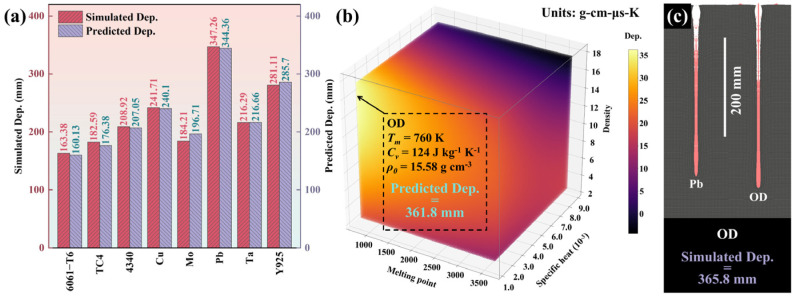
The predicted Dep. and the simulated Dep. of reference materials (**a**); Dep. prediction function within the range of properties of the reference materials (**b**); SCL prediction results and simulation results of ideal material named OD corresponding to the maximum point, and the comparison with Pb SCL simulation result (**c**).

According to the evaluation function, the simulated Dep. and predicted Dep. of the reference materials were compared, as shown in Figure 9a. The evaluation function obtained by quadratic polynomial regression can accurately predict the simulation results. Figure 9b shows the image of the evaluation function of the Dep. within $T_{m}$, $C_{V}$, and $\rho_{0}$ ranges of the reference materials, which visually describes the effects of the three properties on the penetration ability of the SCL. By determining the maximum point of the evaluation function, a theoretical high-penetration material, named OD, within the $T_{m}$, $C_{V}$, and $\rho_{0}$ ranges of the reference materials was obtained. The $T_{m}$, $C_{V}$, and $\rho_{0}$ of OD are 760 K, 124 J kg^−1^ K^−1^, and 15.58 g cm^−3^, respectively. The simulated penetration depth of OD SCL reached 365.8 mm, which was larger than the simulated penetration results of all reference material SCLs, as shown in Figure 9c.

In order to verify the validity of the evaluation formulas, the results of the two materials—Zr Alloy and Ti Alloy—in experiments are compared. The formulas of jet penetration performance given by the quadratic polynomial regression algorithm were used for evaluating the penetration capability of the Zr Alloy and Ti Alloy.

### 3.4. Experimental Verification

It was difficult to find out the material with a melting point of 760 K, specific heat of 124 J kg^−1^ K^−1^, and density of 15.58 g cm^−3^ in a short period of time. To verify the evaluation function, the penetration test of Zr Alloy was used in the experiment test. Zr Alloy was prepared by alloying zirconium, for which powder metallurgy was used in preparing alloy bulk from metal elements with high density and melting point differences into solid solutions with a uniform distribution of elements. Specifically, it is obtained by thoroughly mixing zirconium powder and niobium powder through ball milling, and then shaped by cold isostatic pressing. Finally, it is sintered at 1473 K under the protection of high-purity argon gas for 6 h. The properties of Zr Alloy are shown in Table 7 [29]. Depending on the quadratic polynomial regression formula given above, the penetration and perforation capabilities of these two materials are compared. The Dep. of SCLs with Zr Alloy and Ti Alloy are 224.56 mm and 136.70 mm, respectively. The Dep. of the SCLs with Zr Alloy were 1.6 times that with Ti Alloy. Additionally, the Dia. of Zr Alloy and Ti Alloy are 10.16 mm and 11.80 mm, respectively. These show that, based on numerical simulation and machine learning, the Zr Alloy liner can form a larger penetration depth than the Ti Alloy liner, and the average perforation diameter decreases slightly.

**Figure 10:**
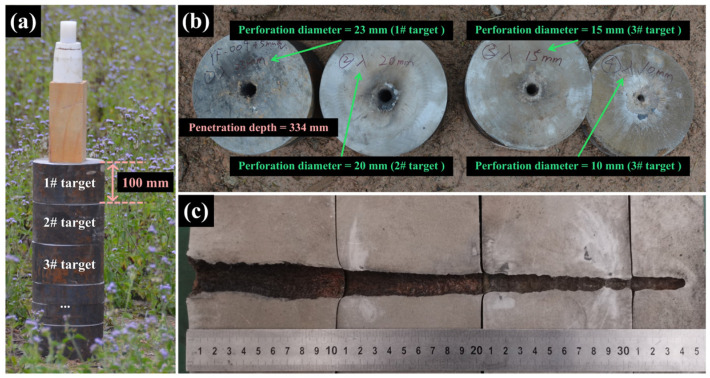
Experimental devices for penetration test of Zr Alloy (**a**), along with the incidence plane (**b**) and longitudinal profile (**c**) of each layer of target after penetration.

The conclusion is verified by experiments. The penetration depth of Zr Alloy SCL was tested using the charge structure shown in Figure 1a and compared with that of Ti Alloy SCL in Figure 4c. As shown in Figure 10a, the jet formed by Zr Alloy SCL driven by RDX explosives penetrated three plates of AISI 1045 with a thickness of 100 mm and formed a crater of about 36 mm on the fourth target. The lateral profile of the penetration hole is shown in Figure 10c, with a total penetration depth of 334 mm, while that of the Ti Alloy SCL is 157 mm, in which the former is 2.1 times greater. The penetration performace of the SCL in experiments was higher than that in predictions from the evaluation function attributed to the shaped charge difference. It should be pointed that the evaluation function used in the prediction of penetration performance has its restriction condition with the present shaped charge structure, but the comparison of the penetration ability among the different material liners should be practicable. The inlets of each target are shown in Figure 10b. The inlet diameter formed by the jet of Zr Alloy (23 mm) is slightly smaller than that of Ti Alloy (between 22 mm and 25 mm), but the difference is slight. This indicates that SCL materials with both penetration and perforation capabilities can be found or designed based on the relative importance ranking and quantitative evaluation methods given above.

In summary, the database formed by reference materials, extension materials, and numerical simulation results was used in the Adaboost algorithm, which pointed out that $T_{m}$, $C_{V}$, and $\rho_{0}$ had the greatest influence on the penetration depth of SCL, while $\rho_{0}$ had a decisive influence on the average perforation diameter of the SCL. The evaluation formulas for the penetration depth and the average perforation diameter of the main material properties were determined by a quadratic polynomial regression algorithm, the effectiveness of which was verified by experiments.

## 4. Analysis and Discussion

### 4.1. Influence Mechanism of Melting Point on Penetration Depth

To disclose the influence mechanism of the selected key properties on the penetration performance of the SCL materials, the SPH algorithm was used for simulating the jet formation and penetration process. As a Lagrange algorithm, the metal jet can be tracked by SPH, with which the temperature change and movement of local material of the SCL were analyzed. Cu is cited as an example, and a three-dimensional SPH model was established to observe the process of jet forming and the penetration of the AISI 1045 target.

**Figure 11:**
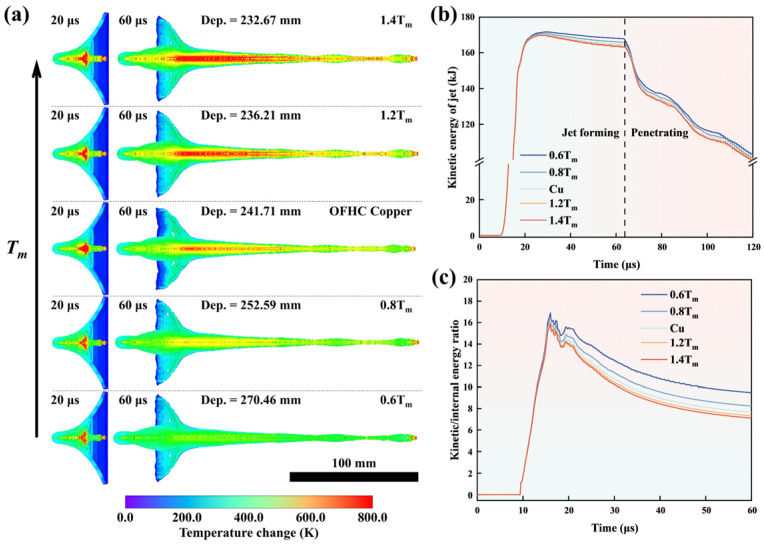
The temperature change fringes of Cu SCLs with different $T_{m}$ at 20 μs and 60 μs after detonation (**a**); The relationships between the kinetic energy of Cu SCLs with different $T_{m}$ and the time after detonation (**b**); The relationships between the ratio of kinetic energy to internal energy of Cu SCLs with different $T_{m}$ and time after detonation (**c**).

$T_{m}$ and $C_{V}$ were the optimized significant properties of the SCL materials in influencing the penetration depth of the SCL. It was considered that the penetration depth can be mediated by the jet temperature and the thermal softening of the SCL material in regulating the $T_{m}$ and $C_{V}$. To clarify the underlying physical mechanisms, the simulation on the SCL of Cu extended materials that was obtained via multipling $T_{m}$ of Cu by 0.6, 0.8, 1.2, and 1.4 was established to analyze the effects of $T_{m}$ on the jet state. Temperature changes caused by adiabatic temperature rise and internal heat conduction of SCL materials with different $T_{m}$ at 20 μs and 60 μs after detonation are shown in Figure 11a. In the first 20 μs, the SCL experienced compression by a detonation wave from the outside to the inside and subsequent formation of the jet head. In combination with Figure 11a,b, it can be seen that the SCL received the kinetic energy transmitted by detonation waves within 20 μs after detonation and gained the adiabatic temperature rise caused by the rapid deformation of the material. This means that, for the first 20 μs, the SCL was loaded with explosives and gained kinetic and internal energy. There is little difference in adiabatic temperature rise and kinetic energy of SCLs with different $T_{m}$ during the compression process of the detonation wave (before 20 μs), as shown in Figure 11b. However, the temperature of the jet formed of the SCL material with a higher $T_{m}$ rose more during the stretching process. Figure 11c shows the relationship of jets with different $T_{m}$ between the kinetic energy–internal energy ratio and time after detonation, which indicates that jets with higher $T_{m}$ converted more kinetic energy into internal energy during the stretch process. In the J-C model, dimensionless temperature ($T^{*}$) is expressed as

(5) $$T^{*}=\frac{T-T_{r}}{T_{m}-T_{r}},$$

where T, $T_{r}$, $T_{m}$ are the material temperature, reference temperature (room temperature), and melting point, respectively [76,87]. The formula states that SCL materials with a lower melting point have better plasticity at the same temperature. At the same initial temperature, SCL materials with a lower $T_{m}$ have less stress at the same strain. The adiabatic temperature rise is

(6) $$∆T=\frac{\beta }{\rho C_{V}}\int_{0}^{\varepsilon_{f}}\sigma \cdot d\varepsilon ,$$

where *β* is the Taylor–Quinney coefficient which characterizes the portion of plastic work converting into heat (ideally, the coefficient is 1.0) [88,89]. It indicates that less stress leads to less temperature rise when the strain process is approximate, thus less kinetic energy is converted into internal energy. This results in a jet with a lower $T_{m}$ carrying a higher residual kinetic energy upon contact with the target and forming a higher penetration depth.

### 4.2. Influence Mechanism of Specific Heat on Penetration Depth

**Figure 12:**
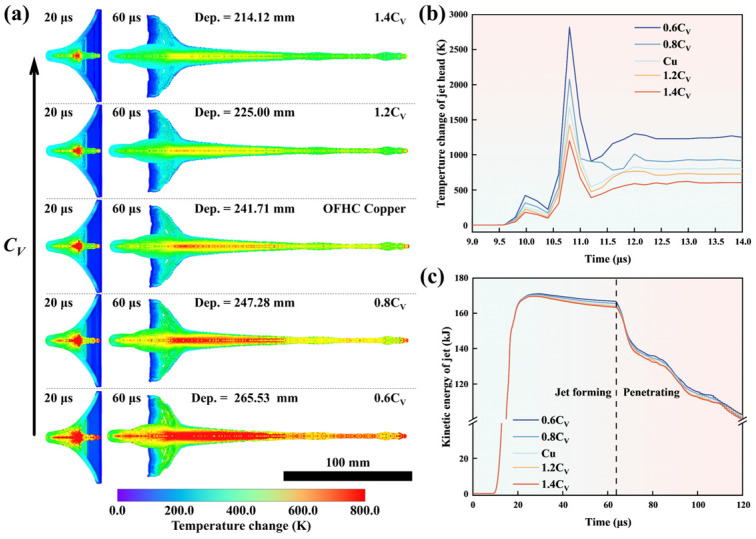
The temperature change fringes of Cu SCL with different $C_{V}$ at 20 μs and 60 μs after detonation (**a**); The relationships between temperature change of jet head with different $C_{V}$ and time after detonation (**b**); The relationships between kinetic energy of jets and time after detonation (**c**).

The influence mechanism of specific heat on penetration depth was investigated. $C_{V}$ of Cu are multiplied by 0.6, 0.8, 1.2, and 1.4 to observe the effects of $C_{V}$ on jet state by the three-dimensional SPH model. According to Figure 12a,c, the morphology and kinetic energy of SCLs with different $C_{V}$ are almost the same at 20 μs. However, an SCL with a lower $C_{V}$ has a higher core temperature, as shown in Figure 12b. According to Formula (6), material with a lower $C_{V}$ can obtain a larger temperature rise in the approximate stress–strain process, which can explain why an SCL with the lower $C_{V}$ has a higher temperature during the compression process of the detonation wave [90]. In the subsequent stretching process, less kinetic energy will be converted into internal energy in the SCL with a lower $C_{V}$, as shown in Figure 12c. According to Formula (5), an SCL with a lower $C_{V}$ has a higher initial temperature, resulting in greater thermal softening effect in the stretching process. The higher kinetic energy of the jet with a lower $C_{V}$ is maintained when the jet contacts the target due to the reduction in the stress–strain integral.

**Figure 13:**
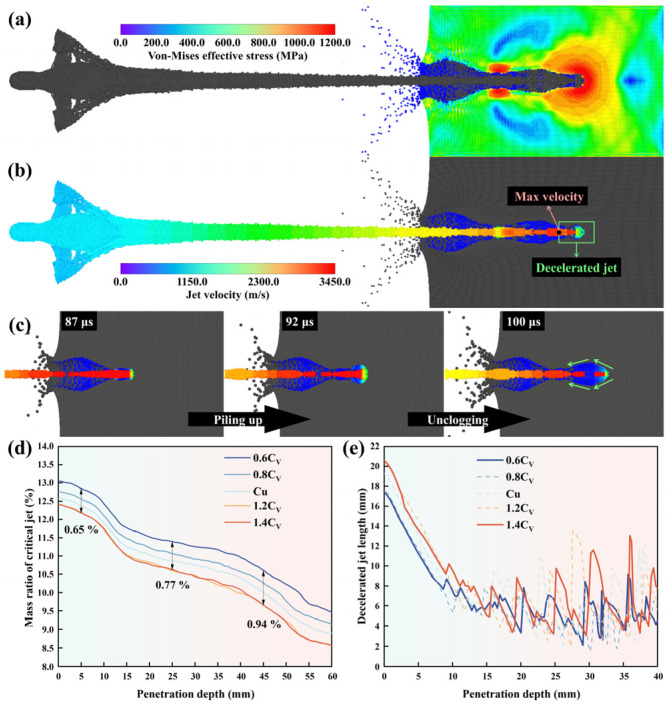
The Von Mises Effective Stress fringe (**a**) of AISI 1045 target and jet velocity fringe (**b**) of Cu SCL during the process of penetration; Piling up and unclogging of jet during the process of penetration (**c**); The relationships between effective jet mass ratio and penetration depth in the process of penetration (**d**); The relationships between head decelerating jet length and penetration depth in the process of penetration (**e**).

In addition, $C_{V}$ also affects the jet penetration process. The state of the jet in the penetration process explains the mechanism of high temperature carried by the jet with a low $C_{V}$ on the penetration process. As shown in Figure 13a, the AISI 1045 target was impacted by the jet and generated stress during the penetration process. At the same time, the jet head was slowed down by the reactive force from the target, and a reverse velocity gradient on itself was generated, as shown in Figure 13b. For the metal jet, the distance between the fastest point and the front end is defined as the decelerated jet length, which can be regarded as the length of the jet piled under the reactive force. The metal jet with the fastest point needs to pierce the decelerated part of the jet to contact the target and form effective penetration. This process is considered as a “self-penetration” or “self-consumption” process [91]. Figure 13c shows the jet piling up and unclogging during the penetration process. According to Figure 13e, during the process of penetration, jets with a lower $C_{V}$ piled up less decelerated jets at the front end. The periodic fluctuation of decelerated jet length with penetration depth verifies the view of the reciprocating action of the decelerated jet piling up and unclogging as shown in Figure 13c. The jets with lower $C_{V}$ have higher temperatures and a greater thermal softening effect, and the decelerated jet is more likely to be unclogged. This means that jets with lower $C_{V}$ have less kinetic energy consumed by the “self-penetration” action, and the effective jet has less loss with increasing penetration depth, as shown in Figure 13d [92].

### 4.3. Influence Mechanism of Density on Penetration Depth

**Figure 14:**
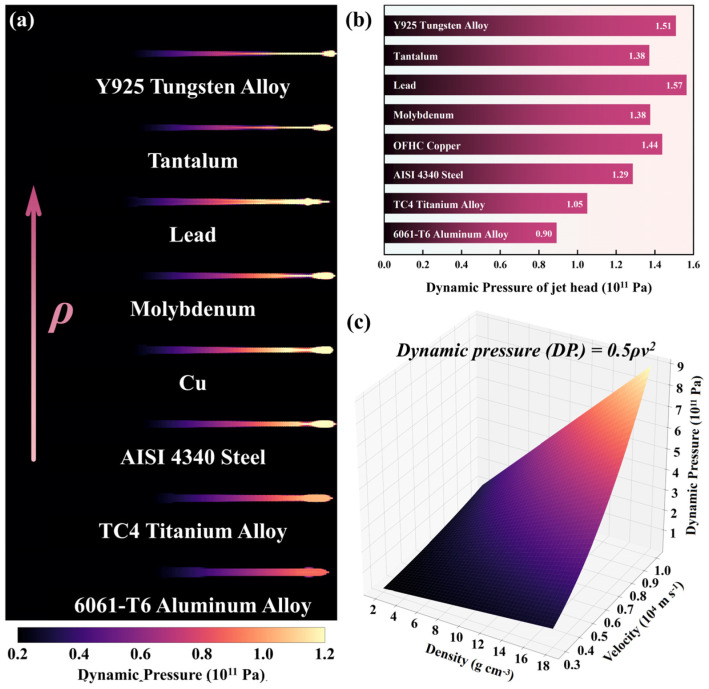
The reference material jet dynamic pressure fringes (**a**) and the jet head dynamic pressures (**b**) at the moment of impact; Dynamic pressure function (**c**) in the range of reference materials’ density and jet velocity.

The two-dimensional ALE model, Python, and LS-Reader were combined for post-processing to analyze the effects of density on the penetration process. The ability of $\rho_{0}$ to influence SCL penetration depth is different in the selected $\rho_{0}$ ranges. As illustrated in Figure 6, within low-$\rho_{0}$ metals (6061-T6, TC4, 4340, and Cu), the velocity of the jet formed by the SCL under explosive loading exhibits a negative correlation with the material density. The accumulation of failure strain in the AISI 1045 target primarily results from the penetration work of the jet. Following the penetration principle of kinetic energy, the dynamic pressure of the jet material can be expressed as

(7) $$DP.=\frac{1}{2}\rho_{0}v^{2},$$

where $\rho_{0}$ is the density assuming the jet is an incompressible fluid and v denotes the speed of the material. The contrast relation of dynamic pressure among reference materials, as shown in Figure 14b, aligns with that of penetration depth, which suggests that dynamic pressure directly influences jet penetration capability. Figure 14a,b illustrate that the metal jet with a low $\rho_{0}$ exhibits a reduced dynamic pressure, which means that the penetration capacity of the jet decreases with decreasing density.

In metal jets with high $\rho_{0}$ (Mo, Pb, Ta, and Y925), the dynamic pressure no longer obviously increases with density. Figure 14c explains the decrease in the dependency of $\rho_{0}$ in dynamic pressure at a high density range. With increasing $\rho_{0}$, the effect of $\rho_{0}$ on dynamic pressure is gradually weakened, while the effect of v gradually strengthens. Jet velocity, not a fundamental property of jet material, is affected by many factors. Consequently, the metal jet with a high density does not show enhanced penetration ability with the increase in density.

### 4.4. Influence Mechanism of Density on Perforation Diameter

**Figure 15:**
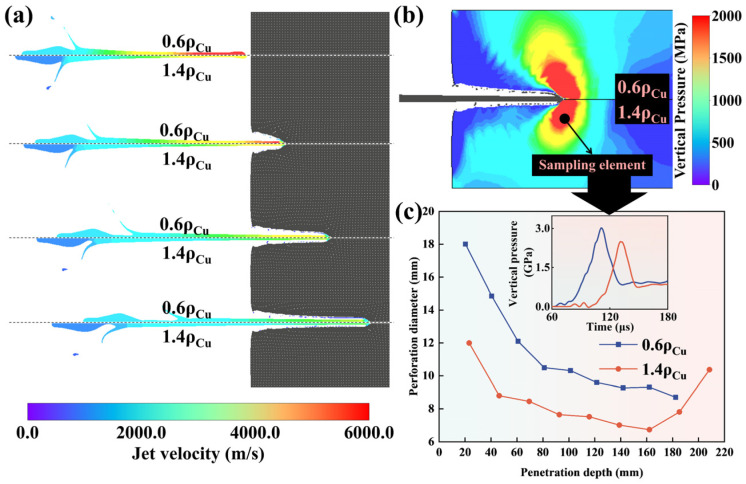
The jet velocity fringes of Cu SCL with different $\rho_{0}$ at the same penetration depth (**a**); Fringes of target vertical pressure (perpendicular to the direction of penetration) in the process of different jets with different $\rho_{0}$ (**b**); The relationships between perforation diameter and penetration depth of jet with different $\rho_{0}$, along with the relationships between vertical pressure of sampling element in target and time after detonation (**c**).

To investigate the effects of density on the perforation process, $\rho_{0}$ of Cu are multiplied by 0.6 and 1.4 by the two-dimensional ALE model to observe the penetration process of the metal jets. The effect of $\rho_{0}$ on perforation capability is significant. The effect of the different jet densities on perforation diameter and penetration depth were compared as shown in Figure 15c. The metal jet with a lower density can form a larger average perforation diameter under the penetration process. The deceleration rate of the jet with a lower density during the penetration is faster, which caused more piling up of the jet material as shown in Figure 15a. This stacking effect increased the lateral effect of the jet, thereby increasing the perforation diameter [10]. As shown by the comparison of vertical pressure of the sampling element at the same penetration depth on the target in Figure 15b,c, the jet with a lower density generated a greater vertical pressure on the target during the penetration process. The stronger lateral effect led to the failure of more target material under vertical pressure [93].

Overall, the penetration depth was dominated by the $T_{m}$ and $C_{V}$ of the SCL that significantly influenced the jet temperature. The SCL with a lower $T_{m}$ can reduce the conversion of kinetic energy to internal energy in the jet stretching process by means of a more significant thermal softening effect. However, the SCL with a lower $C_{V}$ can accumulate a higher temperature during the compression process of the detonation wave, thereby reducing the kinetic energy loss during the subsequent stretching process due to the thermal softening effect. Moreover, the SCL with a lower $C_{V}$ reduces the “self-consumption” of the jet by virtue of a higher jet temperature during the penetration process, which means that the penetration efficiency of the jet is improved. The $\rho_{0}$ of the SCL affects the penetration depth via dynamic pressure of the jet, which causes the dependency of SCL density on the penetration depth to decrease with the increase in density. The jet with lower $\rho_{0}$ can have a greater lateral effect by piling up of jet material, resulting in a larger average perforation diameter.

## 5. Conclusions

By constructing a 2D symmetric ALE-FSI model and combining various machine learning methods (Adaboost regression algorithm and quadratic polynomial regression), the influence of material properties on SCL penetration performance on an AISI 1045 steel target is studied. The main findings are summarized as follows.

(1) Based on the virtual material method, the numerical simulation model for SCL penetration on an AISI 1045 target was established, and the depicted error of the penetration performance is less than 5%. The Adaboost algorithm based on a regression tree pointed out that, among the physical properties described by the J-C model and Mie–Grüneisen EOS, melting point, specific heat, and density have the greatest influence on the penetration depth of the SCL, and their relative importance is 0.4252, 0.3469, and 0.0651, respectively. The density and strain hardening coefficient have the greatest influence on the perforation diameter of the SCL, and the relative importance is 0.7883 and 0.0646, respectively.
(2) The evaluation functions of the melting point, specific heat, and density of SCL materials on penetration depth and perforation diameter were obtained by a quadratic polynomial regression algorithm. An optimally designed SCL material with high penetration performance is given by the maximum point of the evaluation function, from which the key properties are the melting point of 760 K, specific heat capacity of 124 J·kg^−1^·K^−1^, and density of 15.58 g·cm^−3^, respectively. The numerical simulation proves that the SCL of this material can form a theoretical penetration depth of 365.8 cm, which is 51.3% higher than that of OFHC copper.
(3) SCL materials with both excellent penetration and perforation diameter can be found or designed based on the relative importance of the key properties and quantitative evaluation methods. This is proved by the experimental results of the Zr Alloy SCL and Ti Alloy SCL. The penetration depth of Zr Alloy SCL is increased by 114% compared to Ti Alloy SCL, while the inlet diameter is reduced by only about 4%.
(4) The influence mechanisms of the key material properties—density, specific heat, and melting point—on the penetration performace of the SCL were disclosed by tracing the penetration process of the metal jets. Both low specific heat and low melting point can increase the thermal softening effect to reduce the internal work of the SCL under formation and penetration precesses of the metal jet, but low specific heat can also reduce the self-penetrating consumption of the jet during the latter process. Density affects the jet penetration process through influencing dynamic pressure. The penetration depth is significantly increased with density in the SCL of low-density materials, but it is less affected by the density in the high-density materials.

Building upon these findings, we propose that future SCL material design should focus on developing alloys with low melting points, low specific heat capacity, and optimal density. Such materials could simultaneously enhance penetration performance while overcoming the traditional trade-off between penetration depth and perforation capability, representing a promising direction for advanced SCL development.

## Figures and Tables

**Table 1:** JWL EOS parameters of RDX.

Material	ρ_0_ (kg m^−3^)	D (m s^−1^)	A (GPa)	B (GPa)	r1	r2	P_cj_ (GPa)	E (kJ m^−3^)	ω
RDX	1700	8318	854.5	20.5	4.6	1.35	29.5	8.5 × 10^6^	0.25

**Table 2:** J-C model parameters and physical properties of Ti Alloy.

Material	*A* (MPa)	*B* (MPa)	*n*	*C*	${\dot{\varepsilon }}_{0}$ (S^−1)^	*m*	*C_V_* (J kg^−1^ K^−1^)	*T_m_* (K)
Ti Alloy	915	497	0.89	0.012	0.001	0.65	1281	1900

**Table 3:** Mie–Grüneisen EOS parameters of Ti Alloy.

Material	*ρ*_0_ (kg m^−3^)	*C*_0_ (m s^−1^)	*S*_1_	*γ*_0_
Ti Alloy	4700	5130	1.03	1.23

**Table 4:** J-C model parameters and physical properties of AISI 1045.

Material	*A* (MPa)	*B* (MPa)	*n*	*C*	${\dot{\varepsilon }}_{0}$ (S^−1)^	*m*	*C_V_* (J kg^−1^ K^−1^)	*T_m_* (K)
AISI 1045	507	320	0.28	0.064	1	1.06	477	1723

**Table 5:** Mie–Grüneisen EOS parameters of AISI 1045.

Material	*ρ*_0_ (kg m^−3^)	*C*_0_ (m s^−1^)	*S*_1_	*γ*_0_
AISI 1045	7800	4610	1.73	1.67

**Table 6:** Properties of reference materials.

MAT	*ρ*_0_ (10^3^)	*C*_0_ (10^3^)	*S*_1_	*γ*_0_	*A* (10^6^)	*B* (10^6^)	*n*	*C*	*m*	*C_V_*	*T_m_*
6061-T6 Aluminum Alloy	2.70	5.2	1.4	2.0	324	114	0.4	0.002	1.3	876	878
TC4 Titanium Alloy	4.43	5.1	1.0	1.2	1135	250	0.2	0.032	1.1	580	1878
AISI 4340 Steel	7.83	4.6	1.5	2.2	792	510	0.3	0.014	1.0	477	1793
OFHC Copper	8.96	3.9	1.5	2.0	90	292	0.3	0.025	1.1	383	1381
Molybdenum	10.20	5.1	1.3	1.6	425	398	0.6	0.095	0.4	251	3660
Lead	11.27	2.0	1.6	2.8	24	300	1.0	0.100	1.0	124	760
Tantalum	16.65	3.4	1.2	1.6	204	1470	0.8	0.093	0.4	140	3269
Y925 Tungsten Alloy	17.70	4.0	1.2	1.5	631	1258	0.1	0.014	0.9	150	1723

(Units: kg-m-s-K).

**Table 7:** Properties of Zr Alloy and Ti Alloy [2,29].

**MAT**	***ρ*_0_** **(10^3^)**	***C*_0_** **(10^3^)**	***S*_1_**	***γ*_0_**	***A*** **(10^6^)**	***B*** **(10^6^)**	***n***	**C**	${\dot{\varepsilon }}_{0}$ **(S^−1^)**	***m***	***C_V_***	***T_m_***
Zr Alloy	7.0	3.90	1.10	1.20	400	581	0.41	0.039	0.001	1.10	251	2000
Ti Alloy	4.7	5.13	1.03	1.23	915	497	0.89	0.012	0.001	0.65	1281	1900

(Units: kg-m-s-K).

## Data Availability

The original contributions presented in this study are included in the article/Supplementary Materials. Further inquiries can be directed to the corresponding authors.

## Supplementary Materials

The following supporting information can be downloaded at: https://www.mdpi.com/article/10.3390/ma18122742/s1, Table S1: Performance parameters of 6061-T6 Aluminum Alloy and its extension materials; Figure S1: The Python code for the Adaboost algorithm to perform machine learning on DEP. and DIA. datasets; Figure S2: Comparison of properties of Zr Alloy and Ti Alloy.
