# Supplementary material for: Revealing the Influence of Material Properties of Shaped Charge Liner on Penetration Performance via Numerical Simulation and Machine Learning
Source: Materials (Basel). 2025 Jun 11;18(12):2742. doi: 10.3390/ma18122742 (PMC12194528; doi:10.3390/ma18122742)
Supplement: Supplementary file 1 [file materials-18-02742-s001.zip › materials-3659446-supplementary.pdf]

materials

Supporting Information

# Revealing the Influence of Material Properties of Shaped Charge Liner on Penetration Performance via Numerical Simulation and Machine Learning

**Yan Wang [1], Jinxu Liu [1,2,*], Xingwei Liu [1,*], Xinya Feng [1], Yifan Du [1] and Jie Cao [1]**

1 School of Materials Science and Engineering, Beijing Institute of Technology, Beijing 100081, China; wangyanbit@126.com (Y.W.); xinya_feng@126.com (X.F.); duyifan2025@126.com (Y.D.); wy2055917886@163.com (J.C.)
2 China National Key Laboratory of Science and Technology on Materials Under Shock and Impact, Beijing Institute of Technology, Beijing 100081, China
* Correspondence: liujinxu@bit.edu.cn (J.L.); xwliu@bit.edu.cn (X.L.)

## Section S1. The method of obtaining virtual extension materials by changing a single property

Taking the 6061 Aluminum Alloy in part 1 of supporting information as an example, all properties, except $\rho_0$, were multiplied by 0.5 and 1.5, respectively, as shown in Table S1. Based on each reference material, 20 kinds of extension materials were obtained.

**Table S1.** Performance parameters of 6061-T6 Aluminum Alloy and its extension materials.

| MAT | $\rho_0$ ($10^3$) | $C_0$ ($10^3$) | $S_1$ | $\gamma_0$ | A ($10^6$) | B ($10^6$) | n | C | m | $C_V$ | $T_m$ |
|---|---|---|---|---|---|---|---|---|---|---|---|
| **6061-T6** | 2.703 | 5.2 | 1.4 | 2 | 324 | 114 | 0.4 | 0.002 | 1.3 | 876 | 878 |
| **0.5×$C_0$** | 2.703 | **0.262** | 1.4 | 2 | 324 | 114 | 0.4 | 0.002 | 1.3 | 876 | 878 |
| **1.5×$C_0$** | 2.703 | **0.786** | 1.4 | 2 | 324 | 114 | 0.4 | 0.002 | 1.3 | 876 | 878 |
| **0.5×$S_1$** | 2.703 | 5.2 | **0.7** | 2 | 324 | 114 | 0.4 | 0.002 | 1.3 | 876 | 878 |
| **1.5×$S_1$** | 2.703 | 5.2 | **2.1** | 2 | 324 | 114 | 0.4 | 0.002 | 1.3 | 876 | 878 |
| **0.5×$\gamma_0$** | 2.703 | 5.2 | 1.4 | **1** | 324 | 114 | 0.4 | 0.002 | 1.3 | 876 | 878 |
| **1.5×$\gamma_0$** | 2.703 | 5.2 | 1.4 | **3** | 324 | 114 | 0.4 | 0.002 | 1.3 | 876 | 878 |
| **0.5×A** | 2.703 | 5.2 | 1.4 | 2 | **162** | 114 | 0.4 | 0.002 | 1.3 | 876 | 878 |
| **1.5×A** | 2.703 | 5.2 | 1.4 | 2 | **486** | 114 | 0.4 | 0.002 | 1.3 | 876 | 878 |
| **0.5×B** | 2.703 | 5.2 | 1.4 | 2 | 324 | **57** | 0.4 | 0.002 | 1.3 | 876 | 878 |
| **1.5×B** | 2.703 | 5.2 | 1.4 | 2 | 324 | **171** | 0.4 | 0.002 | 1.3 | 876 | 878 |
| **0.5×n** | 2.703 | 5.2 | 1.4 | 2 | 324 | 114 | **0.2** | 0.002 | 1.3 | 876 | 878 |
| **1.5×n** | 2.703 | 5.2 | 1.4 | 2 | 324 | 114 | **0.6** | 0.002 | 1.3 | 876 | 878 |
| **0.5×C** | 2.703 | 5.2 | 1.4 | 2 | 324 | 114 | 0.4 | **0.001** | 1.3 | 876 | 878 |
| **1.5×C** | 2.703 | 5.2 | 1.4 | 2 | 324 | 114 | 0.4 | **0.003** | 1.3 | 876 | 878 |
| **0.5×m** | 2.703 | 5.2 | 1.4 | 2 | 324 | 114 | 0.4 | 0.002 | **0.7** | 876 | 878 |
| **1.5×m** | 2.703 | 5.2 | 1.4 | 2 | 324 | 114 | 0.4 | 0.002 | **2.0** | 876 | 878 |
| **0.5×$C_V$** | 2.703 | 5.2 | 1.4 | 2 | 324 | 114 | 0.4 | 0.002 | 1.3 | **438** | 878 |
| **1.5×$C_V$** | 2.703 | 5.2 | 1.4 | 2 | 324 | 114 | 0.4 | 0.002 | 1.3 | **1313** | 878 |
| **0.5×$T_m$** | 2.703 | 5.2 | 1.4 | 2 | 324 | 114 | 0.4 | 0.002 | 1.3 | 876 | **439** |
| **1.5×$T_m$** | 2.703 | 5.2 | 1.4 | 2 | 324 | 114 | 0.4 | 0.002 | 1.3 | 876 | **1316** |

(Units: kg-m-s-K).

 https://doi.org/10.3390/ma18122742

## Section S2. The Python code for the Adaboost algorithm to perform machine learning on DEP. and DIA. datasets

The hyperparameter grid is embedded in the algorithm to search for the optimal combination of hyperparameters. Lines 23 to 42 optimize the hyperparameters using RandomizedSearchCV.

```python
1 from sklearn.ensemble import AdaBoostRegressor
2 from sklearn.model_selection import train_test_split, RandomizedSearchCV
3 from sklearn.tree import DecisionTreeRegressor
4 from sklearn.metrics import mean_squared_error, mean_absolute_error
5 from sklearn.impute import SimpleImputer
6 import numpy as np
7 import pandas as pd
8 import matplotlib.pyplot as plt
9 from scipy.stats import randint, uniform
10
11 data = pd.read_excel('test.xlsx')
12 X = data.iloc[:, :-2]
13 y1 = data.iloc[:, -2]
14
15 imputer = SimpleImputer(strategy='mean')
16 X = pd.DataFrame(imputer.fit_transform(X), columns=X.columns)
17
18 X_train, X_test, y_train, y_test = train_test_split(X, y1, test_size=0.3, random_state=42)
19
20 base_tree = DecisionTreeRegressor(random_state=42)
21 adaboost = AdaBoostRegressor(base_estimator=base_tree, random_state=42)
22
23 param_dist = {
24     'n_estimators': randint(50, 300),
25     'learning_rate': uniform(0.01, 1.0),
26     'base_estimator__max_depth': randint(3, 10),
27     'base_estimator__min_samples_split': randint(2, 20),
28     'base_estimator__min_samples_leaf': randint(1, 10)
29 }
30
31 random_search = RandomizedSearchCV(
32     adaboost,
33     param_distributions=param_dist,
34     n_iter=200,
35     cv=5,
36     scoring='r2',
37     random_state=42,
38     n_jobs=-1
39 )
40 random_search.fit(X_train, y_train)
41
42 best_adaboost = random_search.best_estimator_
43
44 print("Best Parameters:", random_search.best_params_)
45
46 importance = best_adaboost.feature_importances_
47 features = X.columns
48 indices = np.argsort(importance)[::-1]
49
50 print("\nFeature Importance Values:")
51 for i in indices:
52     print(f"{features[i]}: {importance[i]:.4f}")
53
54 plt.figure(figsize=(10, 6))
55 plt.title("Feature Importances")
56 plt.barh(range(len(indices)), importance[indices], align='center')
57 plt.yticks(range(len(indices)), [features[i] for i in indices])
58 plt.gca().invert_yaxis()
59 plt.xlabel('Relative Importance')
60 plt.show()
61
62 y_pred = best_adaboost.predict(X_test)
63 mse = mean_squared_error(y_test, y_pred)
64 rmse = np.sqrt(mse)
65 mae = mean_absolute_error(y_test, y_pred)
66
67 print(f"\nMSE: {mse:.2f}, RMSE: {rmse:.2f}, MAE: {mae:.2f}")
68 print(f"Train R²: {best_adaboost.score(X_train, y_train):.2f}")
69 print(f"Test R²: {best_adaboost.score(X_test, y_test):.2f}")
```

**Figure S1.** The Python code for the Adaboost algorithm to perform machine learning on DEP. and DIA. datasets.

## Section S3. Radar chart of performance comparison between Zr Alloy and Ti Alloy

The performance parameters of Zr Alloy and Ti Alloy are Compared in Fig. S2. Zr Alloy and Ti Alloy have advantages and disadvantages in $T_{\mathrm{m}}$, $C_{\mathrm{V}}$, $\rho_0$ and $B$ respectively.

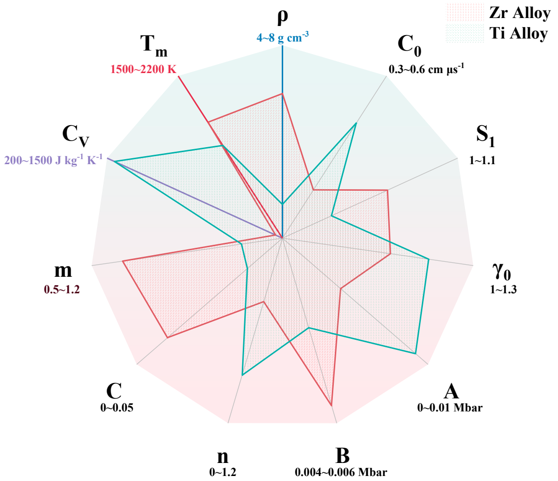


**Figure S2.** Comparison of properties of Zr Alloy and Ti Alloy.
